# Health Care Workers' Adherence to Hand Hygiene Guidelines in Emergency Surgical Room of a Tertiary Care Hospital

**DOI:** 10.1055/s-0042-1749426

**Published:** 2022-06-30

**Authors:** Suchin Sudhakar Dhamnaskar, Gautami Milind Chaudhari, Mandar Sharadchandra Koranne

**Affiliations:** 1 Department of General Surgery, Seth GS Medical College and KEM Hospital, Mumbai, Maharashtra, India

**Keywords:** hand hygiene moments, compliance, emergency surgical room

## Abstract

**Background** Out of every 100 hospitalized patients, 7 patients in advanced countries and 10 patients in emerging countries acquire health care-associated infections (HCAIs). Hand hygiene (HH) procedures are the simple and cost-effective solution to significantly reduce HCAI. We wanted to know the compliance rate of HH procedures among health care workers (HCWs) working in emergency surgical room (ESR) of our institute, so that feedback can be given to them and further interventions can be planned.

**Methodology** This is a cross-sectional observational study conducted in ESR. Resident doctors and faculties, interns, and nurses were directly observed for all the five moments of HH recommended by World Health Organization (WHO). The data have been recorded with the WHO recommended form for observation and basic compliance calculation for HH.

**Results** In total, 1,370 HH opportunities were observed and recorded, of which 690 were for resident doctors and faculties, and 340 each for interns and nurses. The overall total HH compliance rate among all HCWs was 41.3% and resident doctors and faculties had the poorest compliance. Poorest compliance was observed for moment 1, whereas maximum compliance was for moment 3 among all the HCWs.

**Conclusion** HCWs' adherence to HH guidelines in ESR of this tertiary care hospital is low and is least in resident doctors and faculties.

Infections that first appear 48 hours or more after hospitalization or within 30 days after having received health care are called health care-associated infections (HCAIs). 1 2 Out of every 100 hospitalized patients, 7 patients in advanced countries and 10 patients in emerging countries acquire HCAI. 1 3

Hand hygiene (HH) plays an important role in reducing HCAI and spread of antimicrobial resistance. HH is the most cost-effective solution available till date. HH is not only like a “do it yourself” vaccine as described by the Centers for Disease Control and Prevention preventing us from spreading wound infections, respiratory illnesses, diarrheal disorders, etc. but also recognized as the single most important factor in reducing and preventing HCAI.

The most frequently surveyed type of HCAI in developing countries is the risk for patients to develop surgical site infection. This risk is significantly higher in developing countries than in developed countries (e.g., 30.9% in a pediatric hospital in Nigeria, 23% in general surgery in a hospital in the United Republic of Tanzania, and 19% in a maternity unit in Kenya). 4 5 6

There is evidence that adhering to guidelines reduces infections, and still individual clinician adherence to safe HH practices is low worldwide. 7 8 9 10 11

All this formed a background for undertaking the study and wanting to find out the health care workers' (HCWs') adherence to HH guidelines in emergency surgical room (ESR) of our institute.

## Aim

The aim of this study was to find the compliance rate of HH among HCWs in the ESR of a tertiary care hospital.

## Objectives

The objectives of this study were to find the compliance rate toward each moment of HH and to find the compliance rate for each category of HCW.

## Methodology

### Study Design

This is a cross-sectional observational study.

### Study Site

The study was conducted in the ESR of tertiary care hospital. This location was chosen because more HH opportunities are available here due to heavy patient load and high turnover of surgical patients.

### Who Were Observed

Resident doctors and faculties, interns, and nurses were observed. All the HCWs working in the ESR during the expected month of observation part of the study were taken as research participants after taking an informed consent at the beginning of observational period.

### Sample Size

The formula used to calculate the sample size:



where *n* = sample size (HH opportunities)

*p* = adherence quotient, for us, it is the adherence rate of HH

*q* = 100 − *p*

*z* = reliability coefficient (which at confidence interval of 95% is 1.96)

*e* = allowable error (5% of “ *p* ” at confidence interval of 95%)

From the previous literature review, *p* was taken as 40%. 7

So using the above formula,



 (minimum sample size).

Any number of HH opportunities greater than 576.24 can be taken as sample size. To increase the precision of the study, we decided to take higher sample size.

The formula used for this was:

Total sample size = 2 ^*n*^ × minimum sample size

( *n* = number of times the precision of the study is to be increased and minimum sample size here is 576.24)

Hence, total sample size = 2 ^1.25^ × 576.24 = 1,370.

In total, 1,370 HH opportunities were observed and recorded. The opportunities were divided as follows:

1. 690 for resident doctors and faculties
2. 340 for interns
3. 340 for nurses.

The maximum number of HH opportunities was reserved for doctors because previous literature review suggests that HH compliance among doctors is least and it will be more beneficial to get a compliance rate among doctors to create awareness by giving feedback and plan interventional measures.

### What Was Observed

All the five moments of HH recommended by World Health Organization (WHO) were observed. Observation is the “gold standard” for measuring HH adherence according to the WHO guidelines. 8 The only way to directly measure HCWs' adherence to HH guidelines is by observation. 8

A “moment” (indication) is the reason why HH is necessary at a given point of time according to the WHO manual for observers.

WHO's five moments for HH are as follows 8 :

1. Before touching a patient
2. Before clean/aseptic procedure
3. After body fluid exposure/risk
4. After touching a patient
5. After touching patient surroundings.

The points in time within the care process when HH should be performed, as specified by moments (indications) are called “opportunities.” Whenever at least one of the moments (indications) for HH is present and observed, an opportunity exists 12 ; however, there can be more than one moment for a single opportunity. For example, a doctor changes the dressing, removes gloves, and leaves the patient room. The moments (indications) here are (1) after contact with dressings, (2) after removal of gloves, and (3) after contact with the patient. All of these three moments apply to one opportunity or expectation that hands should be cleaned.

“Action” means carrying out the HH procedure. Action should happen at each opportunity. Proper HH actions by HCWs indicate that they are able to identify the moments (indications) during their activities and that they are organizing care within the process. 7

### Observation Method

The data have been recorded with the WHO recommended form for observation and basic compliance calculation for HH.

There was only one observer (who is also the coinvestigator) to collect the data at the study site. The data were collected in sessions of 30 minutes each spanned at different times during the day and night.

The opportunity was not observed, if there was a privacy curtain drawn around the patient's bed. If HH is done at moments which are not indicated as per the WHO recommended five moments of HH, they will be considered as complementary/facultative and will not be recorded by the observer.

*Usage of gloves* : Wearing of gloves, as with all other personal protective equipment, does not compromise the five moments approach to HH. If a moment for HH occurs, it should be complied with. 8

### Statistical Analysis Plan

According to the details provided in the WHO recommended observation form:

1. The overall total compliance rate was calculated in percentage by the formula:

Compliance (%) = (Actions observed/opportunities available) × 100

2. The compliance rate toward each moment was calculated as follows (item by item compliance rate):

Compliance toward moment 1 (%) = (Number of observed HH actions before patient contact/number of HH opportunities observed before patient contact) × 100

Compliance rate toward rest of the moments was calculated similarly.

3. Compliance rate for each category of HCW was also calculated by using the formula:

Compliance for the category of HCW (%) = (Actions observed for the category of HCW/opportunities available for the category of HCW) × 100

Data analysis has been conducted using SPSS version 16.0 data software. Institutional ethics committee approval was taken before start of the study.

## Results

The overall total HH compliance rate among all HCWs was 41.3% and resident doctors and faculties had the poorest compliance. The compliance for moment 1 among all the HCWs together was 33% with resident doctors and faculties having the poorest compliance. The compliance for moment 4 among all the HCWs together was 40.3% with resident doctors and faculties and interns showing poorer compliance than nurses. The compliance for moments 2, 3, and 5 among all the HCWs together was 36.5, 48.7, and 37.6%, respectively, with no significant difference among the HCWs' performance.

**Fig. 1:**
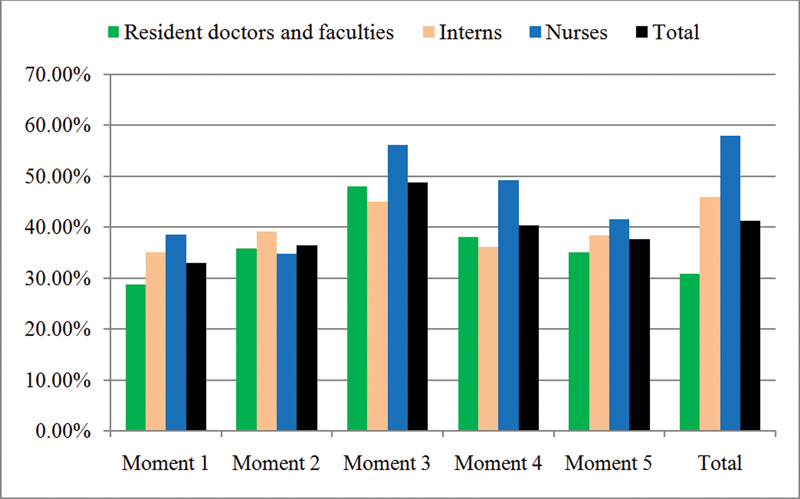
Summary of results ( *x* -axis—compliance rate and *y* -axis—hand hygiene moments).

All these results are summarized in Table 1 and Fig. 1 .

**Table 1:** Summary of results

	Resident doctors and faculties	Interns	Nurses	Total
Moment 1	28.8%	35.1%	38.6%	33%
Moment 2	35.9%	39.1%	34.7%	36.5%
Moment 3	48%	45%	56.1%	48.7%
Moment 4	38.1%	36.1%	49.2%	40.3%
Moment 5	35%	38.4%	41.5%	37.6%
Total	30.9%	45.9%	57.9%	41.3%

## Discussion

According to a study by Onyedibe et al, conducted in Northern Nigeria in February 2020, overall compliance was 31% where a total of 406 HH opportunities were observed among 175 HCWs. 13 Here, compliance was highest after body fluid exposure risk (63%) and was 15.4% in medical doctors and 24% in nurses. Various observational studies of HH adherence conducted between 1981 and 2000 suggest that HCWs' adherence to recommended HH procedures has been poor, with mean baseline rates of 5 to 81% and overall average of 40%. 7 In a study conducted in 2001, by O'Boyle et al, 1,246 indications for HH were studied and overall adherence rate here was 70%. 14

In our study, overall HH compliance rate among all HCWs was 41.3%.

During morning hours, generally when senior doctors are around, HCWs are more likely to be alert regarding HH. Also, many invasive procedures happen in ESR, prompting HH procedures to be followed consciously. At the same time, many life-threatening and dire emergency conditions are being treated here wherein HCWs are likely to miss the HH procedures. In ESR, due to prolonged working hours, HCWs' physical and mental fatigue comes into play. All these situations might have adversely affected the results. However, as the observation sessions were spanned over different time intervals of the day during the study period, we were able to observe the HCWs during different situations of their duties so as to reduce the bias.

Novoa et al in their study conducted in 2007 titled “Evolution of hand hygiene adherence in a tertiary hospital” observed 1,254 opportunities in 247 staff members. Compliance to HH before patient contact was 12.8%, whereas after patient contact, it was 25.6%. 15 In a study by Bahal et al in 2007: “Hand hygiene compliance - universally better post-contact than pre-contact in healthcare workers in the UK and Australia,” it was found that the HH practice in both the study countries is mainly self-protective rather than a patient safety-centered practice. 16 According to another study by Thompson et al in 1997, 230 staff members washed their hands when indicated in 189 patient interactions where compliance rate was 27% before patient contact, 0% during patient care, and 63% after patient contact. 17

In our study, the maximum compliance has been found for moment 3 (48.7%) which is after procedures causing exposure to body fluid or risk of exposure to body fluids. This suggests that there a common mentality among HCWs to only perform HH maximally if there has been an evident risk of infection to themselves and not very much toward the patients from them. This mentality might probably partially be responsible for the overall poor HH compliance among HCWs along with multiple other reasons such as 8 18 :

*Observed risk factors* —Male sex, doctor status (rather than a nurse), nursing assistant status (rather than a nurse), working during week (rather than weekend), working in an intensive care unit, activities with high risk of cross-transmission, wearing gown/gloves, and high number of HH opportunities per hour of patient care.

*Self-reported factors* —Hand washing agents result in irritation and dryness, lack of soap and paper towels, inconveniently located sinks/shortage of sinks, understaffing/overcrowding, inadequate time, patient needs take priority, HH interferes with HCW relationships with patients, low risk of acquiring infection from patients, beliefs that glove use obviates the need for HH, lack of knowledge of guidelines, not thinking about it/forgetfulness, no role model from colleagues or superiors, skepticism regarding the value of HH, disagreement with the recommendations, and lack of scientific information of definitive impact of improved HH on HCAI rates.

*Additional perceived barriers* —Lack of active participation in HH promotion at individual or institutional level, lack of role model for HH, lack of institutional priority for HH, and lack of institutional safety climate.

In a study conducted in 2007 by Trick et al, 6,948 HH opportunities were observed in the three intervention and one control hospitals, and it was found that in all four hospitals, adherence rates over the study period were 42% in nurses, 39% in physicians, and 20% in others. 19 Rosenthal et al in 2005 studied a total of 4,347 HH opportunities and found that compliance rate was 59.6% in nurses, 30.8% in physicians, and 37.1% in ancillary staff. 8 Wendt et al in 2004 made 2,138 observations where adherence rate was overall higher in nurses (67.9%) than in physicians (57.5%). 20

In our study, nurses were most compliant to HH procedures among all categories of HCWs studied with 57.9% compliance rate as compared with 30.9% in resident doctors and faculties and 45.9% in interns.

Follow-up study to identify the specific institutional causes of poor compliance of HH and subsequent targeted interventional study are likely to help in improving the poor compliance rate using multimodal strategies.

## Limitations of the Study

1. Research participants were aware that they will be observed sometime during observation period and this might have affected the final result because of a change in the HCWs' behavior (Hawthorne effect), but they did not know exactly when they will be observed thus reducing Hawthorne effect to some extent.
2. There were instances when a particular HCW was observed more than once as the sample size is the number of HH opportunities and not the number of HCWs. This may have caused a dominant behavior of HH compliance pattern of these HCWs in the final result.
3. The use of only one observer is a limitation of the study because there is no way to assess how reliably observations occurred.

## Conclusion

HCWs' adherence to HH guidelines in ESR of this tertiary care hospital is low and is least in resident doctors and faculties. This has remained low despite enough evidence of the same in the literature. Drastic measures are needed to improve compliance which may include educating HCWs on HH moments, opportunities and actions, repeated feedback of compliance to HCWs, incentives for compliant HCWs, and finally, punitive measures for noncompliers.

## References

[1] Haque M Sartelli M McKimm J Abu Bakar M Health care-associated infections - an overview Infect Drug Resist 2018 11 2321 2333 30532565 10.2147/IDR.S177247 PMC6245375

[2] Revelas A Healthcare - associated infections: a public health problem Niger Med J 2012 53 02 59 64 23271847 10.4103/0300-1652.103543 PMC3530249

[3] Danasekaran R Mani G Annadurai K Prevention of healthcare-associated infections: protecting patients, saving lives Int J Community Med Public Health 2014 1 01 67 68

[4] Gosling R Mbatia R Savage A Mulligan J A Reyburn H Prevalence of hospital-acquired infections in a tertiary referral hospital in northern Tanzania Ann Trop Med Parasitol 2003 97 01 69 73 12662424 10.1179/000349803125002724

[5] Thanni L O Osinupebi O A Deji-Agboola M Prevalence of bacterial pathogens in infected wounds in a tertiary hospital, 1995-2001: any change in trend? J Natl Med Assoc 2003 95 12 1189 1195 14717475 PMC2594861

[6] Koigi-Kamau R Kabare L W Wanyoike-Gichuhi J Incidence of wound infection after caesarean delivery in a district hospital in central Kenya East Afr Med J 2005 82 07 357 361 16167709

[7] Healthcare Infection Control Practices Advisory Committee; HICPAC/SHEA/APIC/IDSA Hand Hygiene Task Force; Society for Healthcare Epidemiology of America/Association for Professionals in Infection Control/Infectious Diseases Society of America Boyce J M Pittet D Guideline for Hand Hygiene in Health-Care Settings. Recommendations of the Healthcare Infection Control Practices Advisory Committee and the HICPAC/SHEA/APIC/IDSA Hand Hygiene Task Force MMWR Recomm Rep 2002 51(RR-16): quiz CE1–CE41 45 12418624

[8] World Health Organization (WHO) WHO Guidelines on Hand Hygiene in Health Care (Advanced Draft): A Summary Geneva, Switzerland WHO 2006

[9] Lam B C Lee J Lau Y L Hand hygiene practices in a neonatal intensive care unit: a multimodal intervention and impact on nosocomial infection Pediatrics 2004 114 05 e565 e571 15492360 10.1542/peds.2004-1107

[10] Pittet D Compliance with hand disinfection and its impact on hospital-acquired infections J Hosp Infect 2001 48(Suppl A ):S40 S46 11759025 10.1016/s0195-6701(01)90012-x

[11] Rosenthal V D Guzman S Safdar N Reduction in nosocomial infection with improved hand hygiene in intensive care units of a tertiary care hospital in Argentina Am J Infect Control 2005 33 07 392 397 16153485 10.1016/j.ajic.2004.08.009

[12] World Health Organization (WHO) World Alliance for Patient Safety: Manual for Observers Geneva, Switzerland WHO 2006

[13] Onyedibe K I Shehu N Y Pires D Assessment of hand hygiene facilities and staff compliance in a large tertiary health care facility in northern Nigeria: a cross sectional study Antimicrob Resist Infect Control 2020 9 01 30 32046790 10.1186/s13756-020-0693-1 PMC7014740

[14] O'Boyle C A Henly S J Larson E Understanding adherence to hand hygiene recommendations: the theory of planned behavior Am J Infect Control 2001 29 06 352 360 11743481 10.1067/mic.2001.18405

[15] Novoa A M Pi-Sunyer T Sala M Molins E Castells X Evaluation of hand hygiene adherence in a tertiary hospital Am J Infect Control 2007 35 10 676 683 18063133 10.1016/j.ajic.2007.03.007

[16] Bahal A Karamchandani A P Fraise A Mclaws M L Hand hygiene compliance: universally better post-contact than pre-contact in healthcare workers in the UK and Australia British Journal of Infection Control. 2007 8 24 28

[17] Thompson B L Dwyer D M Ussery X T Denman S Vacek P Schwartz B Handwashing and glove use in a long-term-care facility Infect Control Hosp Epidemiol 1997 18 02 97 103 9120250 10.1086/647562

[18] Pittet D Improving compliance with hand hygiene in hospitals Infect Control Hosp Epidemiol 2000 21 06 381 386 10879568 10.1086/501777

[19] Chicago Antimicrobial Resistance Project Trick W E Vernon M O Welbel S F Demarais P Hayden M K Weinstein R A Multicenter intervention program to increase adherence to hand hygiene recommendations and glove use and to reduce the incidence of antimicrobial resistance Infect Control Hosp Epidemiol 2007 28 01 42 49 17230386 10.1086/510809

[20] Wendt C Knautz D von Baum H Differences in hand hygiene behavior related to the contamination risk of healthcare activities in different groups of healthcare workers Infect Control Hosp Epidemiol 2004 25 03 203 206 15061410 10.1086/502378

